# The physiological effects of hypobaric hypoxia versus normobaric hypoxia: a systematic review of crossover trials

**DOI:** 10.1186/s13728-014-0021-6

**Published:** 2015-02-26

**Authors:** Jonny Coppel, Philip Hennis, Edward Gilbert-Kawai, Michael PW Grocott

**Affiliations:** University College London Centre for Altitude Space and Extreme Environment Medicine, UCLH NIHR Biomedical Research Centre, Institute of Sport and Exercise Health, 170 Tottenham Court Road, London, W1T 7HA UK; Integrative Physiology and Critical Illness Group, Clinical and Experimental Sciences, Mailpoint 810, Sir Henry Wellcome Laboratories, Faculty of Medicine, University of Southampton, University Hospital Southampton NHS Foundation Trust, Tremona Road, Southampton, SO16 6YD UK; Anaesthesia and Critical Care Research Unit, University Hospital Southampton NHS Foundation Trust, Mailpoint 27, D Level, Centre Block, Tremona Road, Southampton, SO16 6YD UK; NIHR Southampton Respiratory Biomedical Research Unit, Southampton, SO16 5ST UK

**Keywords:** Normobaric hypoxia, Hypobaric hypoxia, Altitude

## Abstract

Much hypoxia research has been carried out at high altitude in a hypobaric hypoxia (HH) environment. Many research teams seek to replicate high-altitude conditions at lower altitudes in either hypobaric hypoxic conditions *or* normobaric hypoxic (NH) laboratories. Implicit in this approach is the assumption that the only relevant condition that differs between these settings is the partial pressure of oxygen (PO_2_), which is commonly presumed to be the principal physiological stimulus to adaptation at high altitude. This systematic review is the first to present an overview of the current available literature regarding crossover studies relating to the different effects of HH and NH on human physiology. After applying our inclusion and exclusion criteria, 13 studies were deemed eligible for inclusion. Several studies reported a number of variables (e.g. minute ventilation and NO levels) that were different between the two conditions, lending support to the notion that true physiological difference is indeed present. However, the presence of confounding factors such as time spent in hypoxia, temperature, and humidity, and the limited statistical power due to small sample sizes, limit the conclusions that can be drawn from these findings. Standardisation of the study methods and reporting may aid interpretation of future studies and thereby improve the quality of data in this area. This is important to improve the quality of data that is used for improving the understanding of hypoxia tolerance, both at altitude and in the clinical setting.

## Background

Hypoxia research has numerous applications. These include investigating the pathogenesis and developing treatments for medical conditions characterised by hypoxia [1] and acute high altitude illness [2], as well as setting optimum training regimes for athletes [3].

Much hypoxia research has been carried out at high altitude in a hypobaric hypoxia (HH) environment. Such ‘field’ studies present practical and logistical challenges including safety concerns about carrying out invasive procedures in a remote setting. For these reasons, many research teams seek to replicate high-altitude conditions at lower altitudes in either hypobaric hypoxic conditions *or* normobaric hypoxic (NH) laboratories. In these two conditions, the hypoxic dose is calculated by the combination of the various barometric pressures × inspired fraction of oxygen [4]. As emphasised in Conkin's ‘Critique of the equivalent air altitude model’ [5], implicit in this approach is the assumption that the only relevant condition that differs between these settings is the partial pressure of oxygen (PO_2_), which is commonly presumed to be the principal physiological stimulus to adaptation at high altitude [6]. Although this assumption underpins the interpretation of many studies that form the basis of hypoxia physiology, it remains open to question as recently highlighted by Millet et al. [7] and controversy exists relating to the sporadic data in this area with various opinions on the matter as discussed in a recent series of ‘point-counterpoints’ [8]. The notion that HH and NH environments are interchangeable in terms of their effect on physiological responses is not proven.

The practical outcomes of this debate affect a variety of fields. Many national teams in various sports incorporate altitude or hypoxic training into their programmes to aid haematological adaptations [3]. Additionally, armies across the world employ pre-acclimatisation strategies to train troops for deployment at high altitude [9]. This issue also applies to medical research such as therapeutic intermittent hypoxic methods [10] or critical care research into tissue hypoxia [1]. Thus, understanding the different impacts of NH and HH on physiology is important.

The aim of this systematic review is to conduct a comprehensive systematic literature search to address the questions: do humans react differently to HH when compared with NH (when evaluated in studies with a crossover design)?

## Review: methods

### Criteria for considering studies for this review

**Figure 1:**
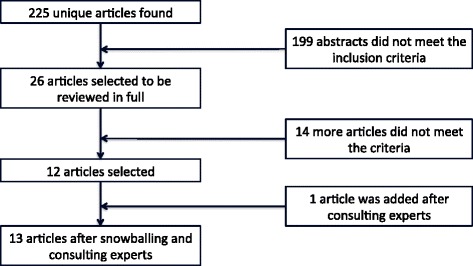
**Methodology flow chart.** This flow chart shows search methodology and results.

Candidate studies were identified using the following criteria (Figure 1).

### Types of studies

We searched for primary research articles describing crossover trials comparing physiological responses to NH and HH. Only crossover trials were considered due to the large inter-subject variation in their response to hypoxia.

### Types of participants

We included studies involving lowland (defined as permanently living in locations <2,000 m) human subjects of any age who were not acclimatised to high altitude.

### Types of interventions

We compared NH and HH. NH and HH must be calculated to be equivalent to the same altitude. We included studies investigating any duration of exposure, and the HH may have been performed either at sea level in a hypobaric chamber or at high altitude.

### Types of outcome measures

Outcome variables were any human physiological response to atmospheric hypoxia. These responses included common phenotypes of interest in high altitude literature including (but not limited to) ventilation, hypoxaemia, exercise metabolism, nitric oxide (NO) production, osmotic balance, erythropoiesis and high-altitude illness.

### Search methods for identification of studies

#### Search strategy

We attempted to identify all relevant trials regardless of language or publication status (published, unpublished, in press, and in progress). A literature search was carried out using the search engines Embase (all to date), Medline (performed on 15 October 2013) and Web of Science (performed on 15 October 2013). Snowballing was carried out; thus, the reference lists of all the shortlisted studies were checked for possible eligible studies.

#### Search terms

Search terms include (‘Hypobari* hypoxia’ OR ‘simulated altitude’ OR ‘hypobari* anoxia’) AND (‘normbari* hypoxia’ OR ‘sea level hypoxia’ OR ‘sea-level hypoxia’ OR ‘normobari* anoxia’).

### Data collection and analysis

#### Selection of studies

Titles and abstracts of candidate studies were screened for eligibility and duplicate references independently by two authors (JC and EG). The reasons for study exclusion were independently documented. For those papers that could not be excluded based on their titles and abstracts, the full paper was read to confirm eligibility. We resolved disagreements by consulting a third author (PH) who arbitrated on inclusion. We obtained the assistance of translators when abstracts were not available in English.

### Data extraction and management

Using data extraction forms, JC extracted information from each study and EG crosschecked the data. Data fields within the data extraction forms were directly linked to the formulated review question and planned assessment of included studies.

The data extraction forms contained the following information: study reference and reviewer identity, verification of study eligibility, study characteristics, study quality (see Quality of data section below), research methods, participants, intervention, outcome measures, results, and additional information.

### Assessment of risk of bias and study quality in included studies

The risk of bias was independently assessed by JC and EG. Studies that do not report statistical significance (*P* values) for reported results were included, but their results were considered either as high risk of bias or unclear. In terms of study quality, we assessed the following: randomisation of subjects for the order of the crossover and whether they were blinded, length of washout period, presence/absence of sample size calculations, whether the statistical analyses accounted for the increased risk of type I errors when analysing large numbers of variables (adjustment for multiple comparisons), test-retest reliability, normalisation of testing environments for humidity (pH_2_O) as this can impact hypoxic dose calculations [11], and control of carbon dioxide (CO_2_) in the testing environments.

### Data synthesis

The results were tabulated and compared. No statistical analysis was carried out because the heterogeneity of the studies was such that the data could not be pooled (see below). Studies were categorised according to duration of hypoxic exposure. One hour was used as the cut off between ‘long’ and ‘short’ studies. All variables identified through our search strategy are highlighted in Table 1 (List of variables) but due to the number of variables reported, not all are considered in the written results and discussion. Emphasis is placed on the major physiological variables and those that are reported in more than one study. This was because a consistent result across multiple independent studies suggests validity of the finding. Additionally, when study characteristics were being determined, if a study did not mention a design feature, such as randomization of exposure order, it was assumed that the feature was not present. The results for each variable and time point can be found in Tables 2, 3, 4, 5, 6.

**Table 1:** **List of variables**

**Oxygenation and ventilation**	**Cardiovascular**	**AMS (acute mountain sickness) and neurology**	**Other**
Ventilation (VE)	Hypoxic cardiac response (HCR)	Postural stability	Sweat rates
Tidal volume (VT)			Forced vascular conductance
		Lake Louise AMS scores	Oesophageal temperature thresholds for increasing forearm skin vascular conductance
Respiratory rate (Bf)	Heart rate variability (LH/HF)		
Exhaled nitric oxide (NO) levels (exNO)			Oesophageal temperature thresholds for increased sweat rate
	Heart rate (HR)		
			Oesophageal temperature
End tidal partial pressure of oxygen (PetO_2_)	Stroke volume (SV)		Skin temperature
			Urine volume
	Cardiac output (CO)		Plasma volume
End tidal partial pressure of carbon dioxide (P_ET_CO_2_)			Glomerular filtration rate (GFR)
			Plasma potassium concentration (plasma K^+^)
			Plasma sodium concentration (plasma Na^+^)
	Blood pressure (BP)		
			Plasma renin activity
			Plasma aldosterone
Alveolar ventilation (VA)			Free water clearance
			Adrenocorticotropic hormone (ACTH)
Volume of CO_2_ produced (VCO_2_)			
			Anti-diuretic hormone (ADH)
			Anti-naturetic protein (ANP)
			Blood base excess
			Urine sodium-potassium ratio (urine Na^+^/K^+^)
Volume of oxygen consumed (VO_2_)			
			Catecholamines, plasma osmolarity
			PH
End tidal fraction of oxygen (FetO_2_)			Urine osmolarity
			Plasma lactate levels
			Blood NO metabolites
			Glutathione peroxidase (GPX)
			MDA
			Nitrotyrosine
End tidal fraction of oxygen (FetCO_2_)			Plasma advanced oxidation protein products and superoxide dismutase
Duration of inspiration and expiration			Haematocrit (Hct)
			Haemoglobin concentration (Hb conc)
Hypoxic ventilatory response (HVR)			
Respiratory quotient (RQ)			
Peripheral oxygen saturations (SpO_2_)			
Arterial oxygen saturations (SaO_2_)			
Arterial oxygen and carbon dioxide partial pressure (P_a_O_2_) (PaCO_2_)			
Arterial oxygen content			
Alveolar-arterial PO_2_ difference			

All the variables measured in the 13 accepted studies are listed. These have been subdivided into physiological systems.

**Table 2:** **Study design**

**Author and year**	**Type of outcome variable**	**Population**	**PiO**_**2**_ **of exposure (calculated by PiO**_**2**_ **= (Pb-47) × FiO**_**2**_ [4]**) (mmHg)**		**Duration of exposure (h:min)**	**Randomised (Y, N)**	**Washout period between trials (days:h)**
			**NH HH**				
Basualto-Alarcon 2012	Ventilatory and cardiovascular + exercise	7 men	3,000 m^c^	3,000 m^c^	00:15 build^a^, 00:10	N	7
Degache 2012	Postural stability	12 men	118 and 102	121 and 103	00:20–25 build^a^, 00:30^b^	Nil stated	<00:24
Hemmingsson 2009	Exhaled NO	6 men, 2 women	103 and 81	99 and 75	00:10 at each altitude	Y	<00:12
Loeppky 2005	Fluid balance	9 men	81	80	00:05 build^a^ 10:00	Y	7
Loeppky 1997*	Ventilatory and cardiovascular	9 men	81	80	00:05 build^a^ 10:00	Y	7
Miyagawa 2010	Ventilatory, cardiovascular and sweat + exercise	7 men	93	97	00:30 build^a^ only for HH 01:40	Y	>6
Naughton 1995	Haematological + exercise	9 subjects with chronic airflow limitation (CAL) with 6 controls	1,829 and 2,438 m^c^	117 and 108	00:12 build^a^ included 00:52:00	Y	00:02
Roach 1996	AMS and cardiovascular	9 men	4,564 m^c^	80	09:00	Y	7
Savourey 2003	Ventilatory and cardiovascular	18 men	4,500 m^c^	4,500 m^c^	00:10 build^a^, 00:30	Y	7
Savourey 2007	Ventilatory and cardiovascular	17 men, 1 woman	4,500 m^c^	4,500 m^c^	00:10 build^a^, 00:30	Y	14
Self 2011	AMS and cardiovascular	17 men and 3 women	7,620 m^c^	7,620 m^c^	00:05	N	<00:24
Tucker 1983	Mixed	11 men	82	80	00:15 build^a^ included 02:20	N	Several weeks
Faiss 2013	Exhaled NO, ventilatory and cardiovascular + exercise	10 men	99	101	24:00:00	Y	23

This table describes all the features of the accepted studies.

^a^When the subjects entered the chamber, the environmental conditions were that of normal sea level but then were gradually made more hypoxic over the specified amount of time until the target hypoxic dose was reached.

^b^The different altitudes were tested consecutively. So sometimes the exposure was 1 h at 3,000 or 1,700 m.

^c^When PiO_2_ could not be calculated due to lack of information, the equivalent attitude estimated by the authors was given.

*P values were for three conditions; in recovery no *P* values unless stated in the discussion.

**Table 3:** **Oxygenation** **and ventilation variables**

**Outcome**	**Author and year**	**Duration of exposure (h:min)**	**Hypobaric hypoxia result [mean (SD)]**	**Normobaric hypoxia result [mean (SD)]**	**Difference (HH − NH)**	***P*** **value**	**Direction of difference NH compared to HH**
VE (L/min)							
	Loeppky 1997	00:00	12.9	13.6	-	-	-
	Savourey 2007	00:10 build, 00:05	10.49 (2.59)	10.14 (1.51)	-	>0.05	NS
	Basualto-Alarcon 2012	00:15 build, 00:05 acclimatisation	10.5 (4.9)	10.3 (1.8)	0.2	-	-
	Basualto-Alarcon 2012	00:15 build, 00:10 acclimatisation	35.7 (5.9)	39.7 (6.7)	-4	<0.05	NH > HH
	Savourey 2003	00:10 build, 00:30	-	-	-	<0.02	NH > HH
	Savourey 2007	00:10 build, 00:30	10.70 (1.93)	10.78 (1.93)	-	>0.05	NS
	Faiss 2013	01:00	13.6 (1.8)	13.3 (3.3)	0.3	>0.05	NS
	Miyagawa 2010^a^	01:05	69.2	65.6	-	>0.05	NS
	Miyagawa 2010	01:10	70.5	65.7	-	>0.05	NS
	Miyagawa 2010	01:20	73.9	70.9	-	>0.05	NS
	Miyagawa 2010	01:40	75.3	77	-	>0.05	NS
	Tucker 1983^b^	02:20 including 00:15 build	2.07	4.82	−2.75	-	-
	Loeppky 1997	03:00	10.3	14.3	−4.0	<0.01	NH > HH
	Loeppky 1997	06:00	10.6	12.7	−2.1	<0.05	NH > HH
	Faiss 2013	08:00	11.8 (1.9)	14.9 (3.5)	−3.1	<0.1	NH > HH
		-	10.7 (1.8)	12.2 (1.6)	−1.5	<0.05	NH > HH
		-	12.7 (2.3)	14.2 (1.5)	−1.5	<0.1	NH > HH
	Loeppky 1997	09:00	10.2	12.2	-	<0.05	NH > HH
	Loeppky 1997	Recovery 12:00:00	9.2	10.1	-	>0.05	NS
VT (L)							
	Savourey 2007	00:10 build^c^, 00:05	0.72 (0.25)	0.88 (0.22)	-	0.03	NH > HH
	Basualto-Alarcon 2012	00:15 build^c^, 00:05 acclimatisation	0.81 (0.36)	0.82 (0.21)	−0.01	-	-
	Basualto-Alarcon 2012	00:15 build^c^, 00:10 acclimatisation	1.85 (0.56)	1.91 (0.53)	−0.06	-	NH > HH
	Savourey 2003	00:10 build^c^, 00:30	-	-	-	<0.001	NH > HH
	Savourey 2007	00:10 build^c^, 00:30	0.83 (0.37)	0.86 (0.34)	-	>0.05	NS
	Faiss 2013	01:00	0.88 (0.21)	0.89 (0.26)	−0.01	>0.05	NS
	Tucker 1983^b^	02:20 including 00:15 build^c^	106	152	-46	-	NH > HH
	Faiss 2013	08:00	0.75 (0.21)	0.94 (0.3)	−0.19	<0.05	NH > HH
	Faiss 2013	16:00	0.75 (0.23)	0.84 (0.24)	−0.9	<0.1	NH > HH
	Loeppky 1997	10:00	-	-	-	>0.05	NS
	Faiss 2013	24:00:00	0.86 (0.25)	0.95 (0.23)	−0.09	<0.05	NH > HH
Bf (cycles/min)							
	Savourey 2007	00:10 build^c^, 00:05	15.73 (4.64)	12.24 (3.80)	-	0.03	HH > NH
	Basualto-Alarcon 2012	00:15 build^c^, 00:05 acclimatisation	13.3 (4.0)	13.4 (4.7)	−0.1	-	-
	Basualto-Alarcon 2012	00:15 build^c^, 00:10 acclimatisation	20.4 (5.4)	22.3 (7.4)	−1.9	<0.05	NH > HH
	Savourey 2003	00:10 build^c^, 00:30	-	-	-	<0.001	HH > NH
	Savourey 2007	00:10 build^c^, 00:30	14.77 (4.17)	13.76 (4.47)	-	>0.05	NS
	Faiss 2013	01:00	16.8 (3.4)	15.9 (4.2)	−0.9	>0.05	NS
	Miyagawa 2010	01:05	31	31	-	>0.05	NS
	Miyagawa 2010	01:10	32	34	-	>0.05	NS
	Miyagawa 2010	01:20	34	36	-	>0.05	NS
	Miyagawa 2010	01:40	38	42	-	>0.05	NS
	Tucker 1983^b^	02:20 including 00:15 build^c^	0.9	3.8	−2.9	-	NH > HH
	Faiss 2013	08:00	16.8 (2.7)	17.1 (4.4)	−0.3	>0.05	NS
	Loeppky 1997	10:00	-	-	-	>0.05	NS
	Faiss 2013	16:00	16.1 (3)	15.8 (3.7)	−0.3	>0.05	NS
	Faiss 2013	24:00:00	16.8 (3.8)	16.2 (3.8)	0.6	>0.05	NS
PetO_2_ (mmHg)							
	Savourey 2007	00:10 build^c^, 00:05	72.5 (6.58)	79.56 (11.94)	-	0.08	Borderline NH > HH
	Savourey 2003	00:10 build^c^, 00:30	-	-	-	>0.05	NS
	Savourey 2007	00:10 build^c^, 00:30	73.15 (7.16)	76.09 (11.61)	-	0.08	Borderline NH > HH
	Faiss 2013	01:00	66.4 (4.1)	62.3 (2.8)	-	4.1	NS
	Tucker 1983^b^	02:20 including 00:15 build^c^	−36.1	−32.6	−3.5	-	-
	Faiss 2013	08:00	61.9 (6.0)	61.6 (2.2)	0.3	>0.05	NS
	Loeppky 1997	10:00	-	-	-	>0.05	NS
	Faiss 2013	16:00	65.0 (5.4)	62.7 (2.6)	2.3	>0.05	NS
	Faiss 2013	24:00:00	65.6 (5.5)	65.6 (2.8)	0	>0.05	NS
PetCO_2_ (mmHg)							
	Savourey 2007	00:10 build, 00:05	44.09 (6.38)	48.87 (5.53)	-	0.05	Borderline NH > HH
	Savourey 2003	00:10 build, 00:30	-	-	-	>0.05	NS
	Savourey 2007	00:10 build, 00:30	43.43 (6.02)	46.13 (6.61)	-	>0.05	NS
	Faiss 2013	01:00	33.4 (2.5)	29.4 (2.4)	4	<0.1	HH > NH
	Tucker 1983^c^	02:20 including 00:15 build	−2.8	−3.6	0.8	>0.05	NS
	Faiss 2013	08:00	33.8 (2.1)	27.5 (1.3)	6.3	<0.01	HH > NH
	Loeppky 1997	10:00	-	-	−1.6	<0.02	NH > HH
	Faiss 2013	16:00	33.1 (1.3)	27.9 (0.9)	5.2	<0.01	HH > NH
	Faiss 2013	24:00:00	30.8 (1.4)	26.5 (1.5)	4.3	<0.01	HH > NH
VA (alveolar ventilation L/min)							
	Loeppky 1997	00:00	9.4	10.1	-	-	-
	Loeppky 1997	03:00	7.2	10.5	−46%	<0.05	NH > HH
	Loeppky 1997	06:00	7.6	9.1	-	<0.05	NH > HH
	Loeppky 1997	09:00	7.6	9.2	-	<0.05	NH > HH
	Loeppky 1997	Recovery 12:00:00	6.7	7.2	-	-	-
VCO_2_ ml/min							
	Loeppky 1997	00:00	295	333	-	-	-
	Miyagawa 2010	01:05	2188	2108	-	>0.05	NS
	Miyagawa 2010	01:10	2121	2007	-	>0.05	NS
	Miyagawa 2010	01:20	2078	2060	-	>0.05	NS
	Miyagawa 2010	01:40	2021	2082	-	>0.05	NS
	Loeppky 1997	03:00	216	330	-	<0.05	NH > HH
	Loeppky 1997	06:00	227	296	-	<0.05	NH > HH
	Loeppky 1997	09:00	235	302	−67	<0.05	NH > HH
	Loeppky 1997	Recovery 12:00	241	267	-	-	-
VO_2_ consumed							
	Loeppky 1997	00:00	329	340	-	-	-
	Miyagawa 2010	01:05	1709	1611	-	>0.05	NS
	Miyagawa 2010	01:10	1783	1637	-	>0.05	NS
	Miyagawa 2010	01:20	1826	1748	-	>0.05	NS
	Miyagawa 2010	01:40	1836	1840	-	>0.05	NS
	Loeppky 1997	03:00	250	361	-	<0.05	NH > HH
	Loeppky 1997	06:00	262	319	-	<0.05	NH > HH
	Loeppky 1997	09:00	278	326	-	<0.05	NH > HH
	Loeppky 1997	12:00	301	291	-	-	-
FetO_2_							
	Savourey 2007	00:10 build^c^, 00:05	-	-	-	>0.05	NS
	Savourey 2003	00:10 build^c^, 00:30	-	-	-	<0.00001	HH > NH
	Savourey 2007	00:10 build^c^, 00:30	-	-	-	>0.05	NS
FetCO_2_							
	Savourey 2007	00:10 build, 00:05	-	-	-	>0.05	NS
	Savourey 2003	00:10 build, 00:30	-	-	-	<0.00001	HH > NH
	Savourey 2007	00:10 build, 00:30	-	-	-	>0.05	NS
Duration of inspiration/s							
	Savourey 2007	00:10 build^c^, 00:05	1.94 (0.65)	2.99 (0.98)	-	0.01	NH > HH
	Savourey 2007	00:10 build^c^, 00:30	2.40 (1.25)	3.00 (1.16)	-	>0.05	NS
Duration of expiration/s							
	Savourey 2007	00:10 build^c^, 00:05	2.09 (0.87)	1.98 (0.84)	-	>0.05	NS
	Savourey 2007	00:10 build^c^, 00:30	-	-	-	>0.05	NS
Hypoxic ventilatory response (HVR) 1%^−1^							
	Savourey 2007	00:10 build^c^, 00:05	−0.05	0.03	-	>0.05	NS
	Savourey 2007	00:10 build^c^, 00:30	−0.09	−0.07	-	>0.05	NS
SpO_2_ (%)							
	Savourey 2007	00:10 build^c^, 00:05	83.03 (4.49)	87.11 (4.81)	−4.08	<0.05	NH > HH
	Basualto-Alarcon 2012	00:15 build^c^, 00:05 acclimatisation	91.6 (4.2)	89.1 (3.8)	2.5	<0.05	HH > NH
	Basualto-Alarcon 2012	00:15 build^c^, 00:10 acclimatisation	85.3 (3.8)	86.0 (1.7)	−0.7	-	-
	Savourey 2003	00:10 build^c^, 00:30	-	-	-	<0.05	NH > HH
	Savourey 2007	00:10 build^c^, 00:30	82.49 (4.39)	85.50 (4.84)	−2.99	0.04	NH > HH
	Faiss 2013	01:00	93 (1)	90 (3)	3	>0.05	NS
	Tucker 1983^b^	02:20 including 00:15 build^c^	−13.2	−13.5	0.3	>0.05	NS
	Faiss 2013	08:00	91 (3)	91 (3)	0	>0.05	NS
	Roach 1996	09:00	83% (1%)	83% (0.7%)	0	>0.05	NS
	Loeppky 2005	10:00	82%	83%	−1%	>0.05	NS
	Loeppky 1997	10:00	-	-	-	>0.05	NS
	Faiss 2013	16:00	92 (2)	91 (2)	1	>0.05	NS
	Faiss 2013	24:00:00	93 (2)	92 (1)	1	>0.05	NS
SaO_2_ (%)							
	Self 2011	00:05	-	-	-	0.005	NH > HH
	Savourey 2007	00:10 build^c^, 00:05	-	-	-	>0.05	NS
	Savourey 2003	00:10 build^c^, 00:30	85% (4)	88% (3)	−3	<0.05	NH > HH
	Savourey 2007	00:10 build^c^, 00:30	81.09% (7.76)	85.48% (5.63)	−4.39	0.07	Borderline NH > HH
	Miyagawa 2010	01:05	82	83	−1	>0.05	NS
	Miyagawa 2010	01:10	81	82	−1	>0.05	NS
	Miyagawa 2010	01:20	81	82	−1	>0.05	NS
	Miyagawa 2010	01:40	82	81	1	>0.05	NS
	Roach 1996	09:00	83% (1%)	83% (0.7%)	0	>0.05	NS
PaO_2_							
	Self 2011	00:05	-	-	-	0.004	HH > NH
	Savourey 2007	00:10 build^c^, 00:05	-	-	-	>0.05	NS
	Savourey 2003	00:10 build^c^, 00:30	6.38 (0.60)	6.90 (0.86)	−0.52	≤0.05	Borderline NH > HH
	Savourey 2007	00:10 build^c^, 00:30	-	-	-	>0.05	NS
CAL subjects	Naughton 1995	00:52	-	-	−1.1	>0.05	NS
Control subjects	Naughton 1995	00:52	-	-	0.7	>0.05	NS
PaCO_2_							
	Self 2011	00:05	-	-	-	0.005	NH > HH
	Savourey 2007	00:10 build, 00:05	-	-	-	>0.05	NS
	Savourey 2003	00:10 build, 00:30	4.65 (0.54)	5.06 (0.46)	−0.41	≤0.05	Borderline NH > HH
	Savourey 2007	00:10 build, 00:30	46.3 (6.5)	52.2 (4.2)	−5.9	0.005	NH > HH
CAL subjects	Naughton 1995	00:52	-	-	0.3	>0.05	NS
Control subjects	Naughton 1995	00:52	-	-	−0.8	>0.05	NS
	Tucker 1983^b^	02:20 including 00:15 build	−3.7	−5.6	1.9	-	-
Alveolar-arterial PO_2_ difference							
CAL subjects	Naughton 1995	00:52	-	-	0.7	>0.05	NS
Control subjects	Naughton 1995	00:52	-	-	0.2	>0.05	NS
Arterial O_2_ content							
	Savourey 2007	00:10 build^c^, 00:05	-	-	-	>0.05	NS
	Savourey 2007	00:10 build^c^, 00:30	-	-	-	>0.05	NS

This table lists all the values of the measured variables that relate to oxygenation and ventilation.

*NS* insignificant, *hyphen* no values given, *CAL* Chronic Airflow Limitation.

^a^Exercise started at 01:00.

^b^Difference from controls.

^c^When the subjects entered the chamber, the environmental conditions were that of normal sea level but then were gradually made more hypoxic over the specified amount of time until the target hypoxic dose was reached.

**Table 4:** **Cardiovascular variables**

**Outcomes**	**Author and year**	**Duration of exposure (h:min)**	**Hypobaric hypoxia result [mean (SD)]**	**Normobaric hypoxia result [mean (SD)]**	**Difference (HH − NH)**	***P*** **value**	**Direction of difference NH compared to HH**
Hypoxic cardiac response (HCR) bpm %−1							
	Savourey 2007	00:10 build^a^, 00:05	−0.61	−0.63	0.02	>0.05	NS
	Savourey 2007	00:10 build^a^, 00:30	−0.52	−0.79	0.27	>0.05	NS
LF/HF%							
	Basualto-Alarcon 2012	00:15 build^a^, 00:10 acclimatisation	1.96 (2.6)	1.28 (0.92)	0.68	<0.05	HH > NH
HR							
	Self 2011	00:01	104.9 (14.3)	96.6 (14.6)	8.3	<0.05	HH > NH
	Self 2011	00:04	-	-	-	>0.05	NS
	Basualto-Alarcon 2012	00:15 build^a^, 00:05 acclimatisation	61 (9)	62 (6)	−1	-	-
	Basualto-Alarcon 2012	00:15 build^a^, 00:10 acclimatisation	129 (23)	134 (16)	−5	<0.05	NH > HH
	Savourey 2007	00:10 build^a^, 00:05	70.32 (9.91)	69.62 (9.95)		>0.05	NS
	Savourey 2003	00:10 build^a^, 00:30	-	-	-	<0.05	HH > NH
	Savourey 2007	00:10 build^a^, 00:30	69.50 (12.07)	70.67 (12.07)	-	>0.05	NS
	Faiss 2013	01:00	62 (8)	63 (10)	−1	>0.05	NS
	Miyagawa 2010	01:05	144	137	-	>0.05	NS
	Miyagawa 2010	01:10	150	146	-	>0.05	NS
	Miyagawa 2010	01:40	166	164	-	>0.05	NS
	Tucker 1983^a^	02:20 including 00:15 build^a^	10.4	3.6	6.8	-	HH > NH
	Faiss 2013	08:00	68 (13)	69 (13)	−1	>0.05	NS
	Faiss 2013	16:00	61 (10)	66 (7)	−5	>0.05	NS
	Faiss 2013	24:00	65 (9)	71 (10)	−6	>0.05	NS
Stroke volume							
	Miyagawa 2010	01:05	105	107	−2	>0.05	NS
	Miyagawa 2010	01:10	113	126	−13	>0.05	NS
	Miyagawa 2010	01:40	116	124	−8	>0.05	NS
Cardiac output							
	Miyagawa 2010	01:05	15.3	14.7		>0.05	NS
	Miyagawa 2010	01:10	17.1	18.2		>0.05	NS
	Miyagawa 2010	01:40	19.4	20.1		>0.05	NS
Mean BP							
	Miyagawa 2010	01:05	112	107		>0.05	NS
	Miyagawa 2010	01:10	108	107		>0.05	NS
	Miyagawa 2010	01:40	100	99		>0.05	NS
BP systolic (Torr)							
	Faiss 2013	01:00	124 (9)	129 (13)	−5	>0.05	NS
	Tucker 1983^b^	02:20 including 00:15 build^a^	−1	5	−6		NH > HH
	Faiss 2013	08:00	124 (9)	123 (7)	1	>0.05	NS
	Faiss 2013	16:00	121 (9)	118 (9)	3	>0.05	NS
	Faiss 2013	24:00:00	131 (10)	129 (9)	2	>0.05	NS

This table lists all the values of the measured variables that relate to the cardiovascular system.

*NS* insignificant.

^a^When the subjects entered the chamber, the environmental conditions were that of normal sea level but then were gradually made more hypoxic over the specified amount of time until the target hypoxic dose was reached.

^b^Difference from controls.

**Table 5:** **AMS and neurology variables**

**Outcome**	**Author and year**	**Duration of exposure (h:min)**	**Hypobaric hypoxia result [mean (SD)]**	**Normobaric hypoxia result [mean (SD)]**	**Difference (HH − NH)**	***P*** **value**	**Direction of difference NH compared to HH**
Length of centre of pressure trajectory in *Y* axis							
Eyes open 1,700 m	Degache 2012	00:30	114.2 (38.8)	129.5 (53.3)	-15.3	-	NH > HH
Eyes closed 1,700 m	Degache 2012	00:30	127.2 (54.9)	87.7 (44.8)	39.5	-	HH > NH
Dual task 1,700 m	Degache 2012	00:30	128.7 (87.1)	79.9 (30.3)	48.8	-	HH > NH
Romberg’s index 1,700 m	Degache 2012	00:30	1.35 (0.19)	1.42 (0.34)	-0.07	-	-
Eyes open 3,000 m	Degache 2012	00:30	123.1 (22.6)	127.2 (41.5)	-4.1	-	-
Eyes closed 3,000 m	Degache 2012	00:30	104.7 (27.0)	89.1 (39.9)	15.6	-	HH > NH
Dual task 3,000 m	Degache 2012	00:30	91.9 (22.4)	82.4 (30.4)	9.5	-	HH > NH
Romberg’s index 3,000 m	Degache 2012	00:30	1.33 (0.22)	1.39 (0.29)	-0.06	-	-
Variance of speed of CoP							
Eyes open 1,700 m	Degache 2012	00:30	111.0 (56.2)	151.4 (30.2)	-40.4	-	NH > NH
Eyes closed 1,700 m	Degache 2012	00:30	111.0 (58.8)	149.9 (31.5)	-38.9	-	NH > HH
Dual task 1,700 m	Degache 2012	00:30	112.1 (57.7)	151.1 (31.7)	-39	-	NH > HH
Romberg’s index 1,700 m	Degache 2012	00:30	0.98 (0.09)	0.99 (0.03)	-0.01	-	-
Eyes open 3,000 m	Degache 2012	00:30	150.5 (42.3)	160.8 (14.0)	-10.3	-	NH > HH
Eyes closed 3,000 m	Degache 2012	00:30	142.9 (40.8)	158.6 (13.7)	-15.7	-	NH > HH
Dual task 3,000 m	Degache 2012	00:30	143.4 (39.2)	160.1 (15.0)	-16.7	-	NH > HH
Romberg’s index 3,000 m	Degache 2012	00:30	0.95 (0.11)	0.99 (0.02)	-0.04	-	-
Lake Louise AMS scores	Self 2011	00:01	-	-	2.36	>0.05	NS
	Self 2011	00:04	-	-	-4.89	>0.05	NS
	Roach 1996	09:00	-	-	-	<0.01	HH > NH
	Loeppky 2005*	10:00	-	-	-	<0.001	HH > NH

This table lists all the values of the measured variables that relate to AMS and neurology.

*NS* insignificant.

*P value calculated including hypobaric normoxia.

**Table 6:** **Additional physiological variables**

**Outcome**	**Author and year**	**Duration of exposure (h:min)**	**Hypobaric hypoxia result [mean (SD)]**	**Normobaric hypoxia result [mean (SD)]**	**Difference (HH − NH)**	***P*** **value**	**Direction of difference NH compared to HH**
Exhaled NO (PE NO)							
	Hemmingsson 2009	00:10 at each ascending altitude	-	-	33% mean reduction (at 5,000 m)	0.002	NH > HH
	Faiss 2013	01:00	9.5 (5.0)	14.9 (9.2)	−5.4	<0.01	NH > HH
	Faiss 2013	08:00	8.8 (5.3)	14.1 (7.4)	−5.3	<0.01	NH > HH
	Faiss 2013	16:00	7.9 (4.5)	14.7 (8.6)	−6.8	<0.01	NH > HH
	Faiss 2013	24:00:00	8.9 (5.4)	15.7 (8.7)	−5.8	<0.01	NH > HH
RQ							
	Self 2011	00:05	2.37 (0.53)	1.41 (0.15)	0.96	0.005	HH > NH
Forced vascular conductance							
	Miyagawa 2010	01:00-01:40	-	-	-	>0.05	NS
Sweat rate							
	Miyagawa 2010	01:00-01:40	-	-	-	>0.05	NS
Oesophageal temperature thresholds for increasing forearm skin vascular conductance							
	Miyagawa 2010	01:00-01:40	-	-	-	>0.05	NS
Oesophageal temperature thresholds for increasing sweat rate							
	Miyagawa 2010	01:00-01:40	-	-	-	>0.05	NS
Oesophageal temperature							
	Miyagawa 2010	01:05	36.63	36.61	0.02	>0.05	NS
	Miyagawa 2010	01:10	37.12	37.11	0.01	>0.05	NS
	Miyagawa 2010	01:40	37.95	37.96	−0.01	>0.05	NS
Skin temperature							
	Miyagawa 2010	01:05	33.37	33.47	−0.1	>0.05	NS
	Miyagawa 2010	01:10	33.35	33.43	−0.08	>0.05	NS
	Miyagawa 2010	01:40	34.44	34.59	−0.15	>0.05	NS
Urine vol (ml)							
	Loeppky 2005^a^	10:00	-	-	-	0.005	HH > NH
	Tucker 1983^b^	02:20 including 00:15 build^d^	−1.6	0.1	−1.7	-	NH > HH
Plasma volume							
	Miyagawa 2010	01:00-01:40	-	-	-	>0.05	NS
	Loeppky 2005	10:00	-	-	−6%	0.002**	HH > NH
GFR							
	Loeppky 2005	10:00	-	-	-	>0.05	NS
Plasma K^+^							
	Loeppky 2005	10:00	-	-	-	0.003	NH > HH
Plasma Na^+^							
	Loeppky 2005	10:00	-	-	-	0.006	NH > HH
Plasma renin activity (PRA)							
	Loeppky 2005^a^	10:00	-	-	-	<0.05	HH > NH
Plasma aldosterone							
	Loeppky 2005^a^	10:00	-	-	-	<0.001	NH > HH
Free water clearance (CH_2_O)							
	Loeppky 2005^a^	10:00	-	-	-	<0.05	HH > NH
ACTH							
	Loeppky 2005^a^	10:00	-	-	-	0.18	NS
ADH							
	Loeppky 2005	10:00	-	-	-	>0.05	NS
ANP							
	Loeppky 2005*	10:00	-	-	-	0.97	NS
Blood base excess							
	Loeppky 2005	10:00	-	-	-	>0.05	NS
Urine Na^+^/K^+^							
	Loeppky 2005	10:00	-	-	-	0.7	NS
Catecholamines							
	Miyagawa 2010	01:00-01:40	-	-	-	>0.05	NS
	Loeppky 2005	10:00	-	-	-	0.43	NS
Haematocrit							
	Miyagawa 2010	01:00-01:40	-	-	-	>0.05	NS
	Tucker 1983^b^	02:20 including 00:15 build^d^	0.7	0.7	0		NS
Haemoglobin concentration							
	Savourey 2007	00:10 build^d^, 00:05	-	-	-	>0.05	NS
	Savourey 2007	00:10 build^d^, 00:30	-	-	-	>0.05	NS
	Miyagawa 2010	01:00-01:40	-	-	-	>0.05	NS
Plasma osmolarity (mOsm)							
	Miyagawa 2010	01:00-01:40	-	-	-	>0.05	NS
	Tucker 1983^b^	02:20 including 00:15 build^d^	−0.4	−1	0.6	-	HH > NH
pH							
	Savourey 2003	00:10 build^d^, 00:30	7.46 (SEM 0.03)	7.44 (SEM 0.02)	0.02	≤0.05	Borderline HH > NH
	Savourey 2007	00:10 build^d^, 00:30	7.45 (0.04)	7.44 (0.04)	0.01	0.02	HH > NH
CAL subjects							
	Naughton 1995	00:52	-	-	−0.02	>0.05	NS
Control subjects							
	Naughton 1995	00:52	-	-	0.01	>0.05	NS
	Tucker 1983^d^	02:20 including 00:15 build^d^	0.047	0.015	0.032	-	HH > NH
	Faiss 2013	24:00:00	-	-	-	<0.01	NH > HH
Urine osmolarity (mOsm)							
	Tucker 1983^c^	02:20 including 00:15 build^b^	15	0.1	14.9	-	HH > NH
Lactate mmol/kgH_2_O							
	Miyagawa 2010	01:00-01:40	-	-	-	>0.05	NS
Blood NO metabolites							
	Faiss 2013	01:00	31.6 (19.6)	27.7 (7.3)	3.9	<0.01	HH > NH
	Faiss 2013	08:00	28.1 (18.9)	32.7 (9.7)	−4.6	<0.01	NH > HH
	Faiss 2013	16:00	24.2 (16.3)	30.2 (7.1)	−6	<0.01	NH > HH
	Faiss 2013	24:00	22.85 (16.2)	28.9 (6.9)	−6.05	<0.01	NH > HH
GPX (% baseline)							
	Faiss 2013	01:00	114 (26)	111 (30)	3	>0.05	NS
	Faiss 2013	08:00	85 (27)	123 (23)	−37	>0.05	NS
	Faiss 2013	16:00	105 (43)	107 (21)	−2	>0.05	NS
	Faiss 2013	24:00	103 (43)	107 (21)	−4	>0.05	NS
MDA (% baseline)							
	Faiss 2013	01:00	117 (40)	92 (36)	25	>0.05	NS
	Faiss 2013	08:00	103 (62)	111 (35)	−8	>0.05	NS
	Faiss 2013	16:00	111 (56)	116 (55)	−5	>0.05	NS
	Faiss 2013	24:00	108 (52)	97 (51)	11	>0.05	NS
Nitrotyrosine (% baseline)							
	Faiss 2013	01:00	86 (16)	105 (26)	−19	>0.05	NS
	Faiss 2013	08:00	77 (35)	75 (37)	2	>0.05	NS
	Faiss 2013	16:00	91 (20)	98 (16)	−7	>0.05	NS
	Faiss 2013	24:00	75 (40)	87 (25)	−12	>0.05	NS
Plasma advanced oxidation protein products							
	Faiss 2013	01:00	120%	13%	107%	-	NH > HH
	Faiss 2013	24:00	260%	88%	172%	-	NH > HH
Superoxide dismutase	Faiss 2013	24:00	-	-	37%	-	NH > HH

This table lists all the values of all other the measured physiological variables.

*NS* insignificant, *SEM* Standard Error of the Mean.

^a^Measured 2 h after exposure.

^b^Difference from controls.

^c^Subjects were gradually exposed increasing levels of hypoxia over the stated time until the target hypoxic dose was reached.

^d^When the subjects entered the chamber, the environmental conditions were that of normal sea level but then were gradually made more hypoxic over the specified amount of time until the target hypoxic dose was reached.

*P value calculated including hypobaric normoxia.

**P value calculated including hypobaric normoxia and after 3 h.

## Review: results

### Studies

A total of 225 unique articles were identified in the EMBASE, MEDLINE and Web of Science searches. After applying our inclusion and exclusion criteria, 13 studies were deemed eligible for inclusion (Table 2: Study design). A total of 153 subjects were included in our review. Of these, six subjects were women and nine had chronic airflow limitations. One study was added after snowballing [12].

The studies investigated simulated altitudes from 1,700 m to 7,620 m, and exposure to the hypoxia lasted between 5 min to 24 h. The 13 studies were carried out in seven different countries: Australia (1), Japan (1), Spain (1), Sweden (1), Switzerland (2), France (2), and America (5).

### Variables measured

#### Quality of data

##### Study design

Nine studies [12-20] randomised the order of the crossover. Three studies [6,21,22] did not, and one [23] was ambiguous as to whether randomization was used or not. Three studies [12,18,22] had a washout period of at least 14 days, six studies [13-17,21] used 7 days, and four studies [6,19,20,23] used less than 24 h.

The largest study involved 20 people, the smallest 7, and the mean was 12. None of the studies stated they had conducted a sample size calculation to justify their chosen number. Only two studies [12,23] mentioned accounting for the inflated risk of type I errors that arises when multiple comparisons are made, and both of these performed Bonferroni adjustments. Only one study measured the test-retest reliability of their outcome variable [23]. They performed an intra-class coefficient correlation and showed a good reliability of the postural stability measurements.

The methods used to control the degree of hypoxia administered in each study varied. Five studies mentioned controlling the relative humidity between HH and NH. Of these, three [15,17,18] maintained 50% humidity (±1%) and two [13,14] maintained it between 45% and 55%. One paper [6] specifically mentioned the measurement and control of CO_2_ levels in the chambers using CO_2_ scrubbers.

### Ventilation

**Figure 2:**
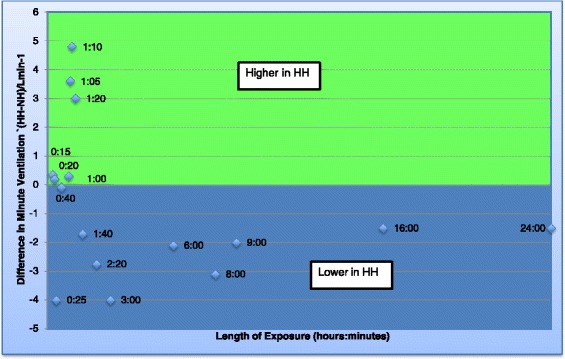
**Graph of minute ventilation.** Graph to show the difference in minute ventilation between the two environments over time. Each data point represents data obtained from a study and the number refers to the time point. If the data point is in the green area, the minute ventilation was found to be higher in HH but if in the blue area, the minute ventilation was found to be lower in HH.

**Figure 3:**
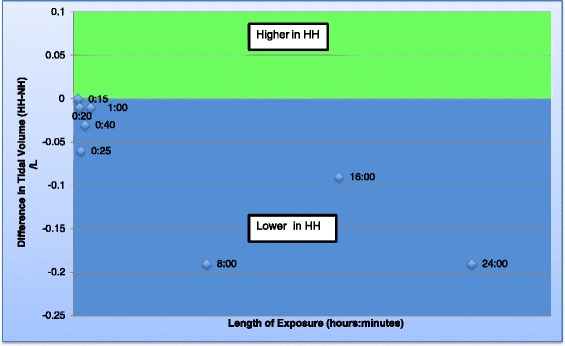
**Graph of tidal volume.** Graph to show the difference in tidal volume between the two environments over time. Each data point represents data obtained from a study and the number refers to the time point. If the data point is in the green area, the tidal volume was found to be higher in HH but if in the blue area, the tidal volume was found to be lower in HH.

**Figure 4:**
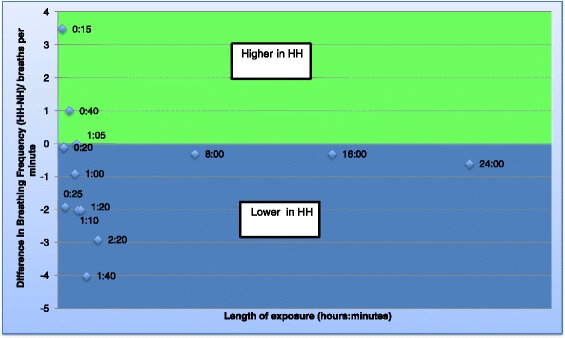
**Graph of breathing frequency.** Graph to show the difference in breathing frequency between the two environments over time. Each data point represents data obtained from a study and the number refers to the time point. If the data point is in the green area, the breathing frequency was found to be higher in HH but if in the blue area, the breathing frequency was found to be lower in HH.

Eight studies were identified that reported ventilation and oxygenation. Five of these lasted ≥1 h (long studies) [12,13,15,22,16], and three lasted <1 h (short studies) [17,18,21]. Five out of seven studies reporting minute ventilation reported values that were significantly lower in HH [12,13,17,21,22] (by up to 4 L/min) [13] (Figure 2: Graph of minute ventilation), whereas two identified no difference between conditions [15,18]. Consistent with this, the tidal volume was lower in HH in five out of six studies where this was reported (Figure 3: Graph of tidal volume) [12,17,18,21,22]. The largest difference in tidal volume found in a study was 0.9 L [12]. Two of the seven studies reporting breathing frequency found it to be higher in HH [17,18], whilst two others reported lower values in HH [21,22] and there was no difference in the remainder [12,13,15] (Figure 4: Graph of breathing frequency). The only study that reported alveolar ventilation found that it was higher in NH than HH [13].

### Oxygenation

The peripheral oxygen saturations measured by pulse oximetry (SpO_2_) were significantly lower in HH in two out of three short studies [17,18]. One study found that the saturations were 4.08% lower in HH [18]. However, no differences were found in all five of the long studies [12-14,16,22]. The arterial blood saturations (SaO_2_) were lower in HH in all three short studies [6,17,18] but not in the two longer studies [15,16]. Arterial partial pressures of oxygen (P_a_O_2_) was lower in NH in one study [6], higher in NH in one study [17], and no different in two studies [18,20] (Table 3: Oxygenation and ventilation). Only the two studies by Savourey et al. [17,18] measured the end tidal fractions of O_2_ and these report discordant results. In 2003, Savourey et al. [17] found the end tidal fractions of O_2_ to be higher (*P* < 0.00001) in HH than NH; however, in their 2007 [18] study following the same protocols, no difference was demonstrated (*P* > 0.05).

### Carbon dioxide (CO_2)_ clearance

In three out of five studies, there was no difference in the end tidal partial pressure of CO_2_ (P_ET_CO_2_) between HH and NH [17,18,22]; however, one study [12] found it to be higher in HH and one found it to be lower in HH [13]. In four out of five studies, the PaCO_2_ levels were lower [11,17,13] or the same [20] in HH compared to NH. For example, one study found the difference in PaCO_2_ to be as large as 5.9 hPa lower in HH [18]. Two studies measured the end tidal fractions of CO_2_ [17,18]. In 2003, Savourey et al. [17] found the end tidal fractions of CO_2_ to be higher (*P* < 0.00001) in HH than NH, whereas in the same group's 2007 study [18] (following the same protocols), no difference was demonstrated (*P* > 0.05).

### Cardiovascular variables

**Figure 5:**
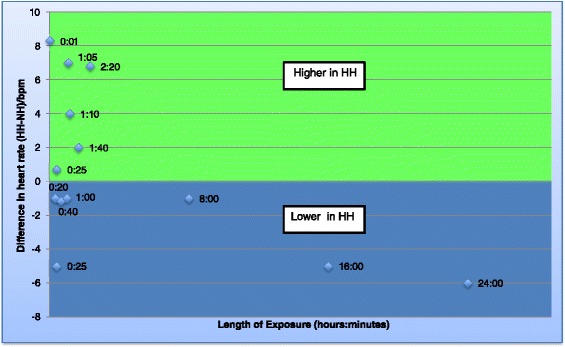
**Graph of heart rates.** Graph to show the difference in heart rate between the two environments over time. Each data point represents data obtained from a study and the number refers to the time point. If the data point is in the green area, the heart rate was found to be higher in HH but if in the blue area, the heart rate was found to be lower in HH.

Seven studies were identified that reported physiological variables relating to the cardiovascular system. Three of these were long studies [12,15,22] and four were short studies [6,17,18,21] (Table 4: Cardiovascular variables). All seven studies measured heart rate (HR). Three found HR to be higher in HH [6,17,22], one found it to be lower [21], and the others found no differences [12,15,18] (Figure 5: Graph of heart rates). Three studies measured blood pressure. Two found no difference in blood pressure between environments [12,15]; however, one other found it to be lower in HH than in NH [22]. Sympathetic drive, measured by a specific electrocardiogram (ECG) recorder of low- and high-frequency components of heart rate, was only investigated in one study [21] and was found to be higher in HH than in NH.

### Acute mountain sickness and neurology

Four studies were found relating to acute mountain sickness (AMS) and neurological symptoms and signs, two of which were long studies [14,16] and two of which were short studies [6,23] (Table 5: AMS and neurology). Two out of three studies [14,16] that measured AMS scores found that AMS was significantly worse in HH than in NH. Only one study [23] measured postural stability, which was significantly reduced in HH compared to NH. Subjects deviated from the midline more in HH than NH, and the speed with which movements occurred to correct their posture was slower.

### Additional physiological variables

**Figure 6:**
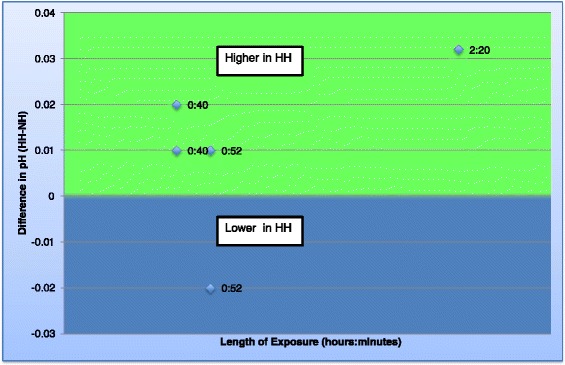
**Graph of pHs.** Graph to show the difference in pH between the two environments over time. Each data point represents data obtained from a study and the number refers to the time point. If the data point is in the green area, the pH was found to be higher in HH but if in the blue area, the pH was found to be lower in HH.

Six studies [6,12,14,15,19,22] researched a variety of other physiological variables (Table 6: Additional physiological variables). In three out of five of these studies [17,18,22], the plasma pH was higher in HH than NH; however, one of the other papers found the pH to be higher in NH [12] (Figure 6: Graph of pH). The greatest difference in pH found in a study was 0.032 [22]. Urine osmolarity was measured in only one study and was significantly higher in HH [22]. Additionally, the same study was the only one to measure the volume of urine produced and found it to be lower during HH exposure [22]. One study also measured K^+^, Na^+^ and aldosterone concentrations in plasma and found they were higher in NH [14]. In recovery post-hypoxic exposure, authors in [14] found that the urine volume, plasma renin activity and free water clearance were higher whilst aldosterone was lower after HH than NH. This was the only study to measure these variables.

Only two studies [12,19] measured exhaled NO levels and both found that they were lower in HH than in NH. The greatest difference in exhaled NO found in these papers was 6.8 mmHg [12]. Additionally, only one study measured systemic NO and the authors found it was lower in HH [12]. The same study was the only study to measure plasma-advanced oxidation protein products and superoxide dismutase. These were higher in HH [12]. Only one study [6] measured the respiratory quotient (RQ) and found it was higher in HH than NH.

## Review: discussion

### Summary of physiological findings

We believe this is the first systematic review comparing the effect of NH and HH on human physiology. Significant differences were demonstrated in variables relating to ventilation, NO, fluid retention, and in factors relating to AMS. For other variables, there was no consistent pattern across the reviewed studies.

#### Oxygenation and ventilation parameters

The majority of studies included data relating to oxygenation and ventilation. Oxygen saturations measured from pulse oximetry (SpO_2_) and arterial blood gases correlated with each other. During short-term exposure, oxygen saturations were lower in HH [6,17]. This was not the case for long-term exposure, perhaps as the initial process of acclimatisation to hypoxia had occurred [18,20]. P_a_O_2_ did not differ at any stage.

A decreased minute ventilation and alveolar ventilation was seen in HH. This finding is in accord with the smaller tidal volumes demonstrated in HH; however, breathing frequencies varied [12,13,17,18,21,22]. Despite the lower SpO_2_ in HH initially, oxygen saturations were maintained at the same level in both environments over longer time periods. Interestingly, despite the reduction in ventilation, P_ET_CO_2_ levels did not change significantly [12,13,17,18,22].

Notably, Savourey et al. found different results in end tidal fractions of both O_2_ and CO_2_ between their two studies in 2003 [17] and 2007 [18]. This not only makes interpretation of their results very challenging but also highlights issues concerning reliability of studies (as discussed in Study quality section).

#### Cardiovascular variables

The majority of these variables were similar for HH and NH. Heart rate and blood pressure results were inconsistent, although some data suggests that heart rate may be raised initially in HH [6,12,15,17,18,21,22].

#### AMS and neurology variables

Mountain sickness is an area of research that has attracted much interest and consequently the multiple variables that combine to make the Lake Louise AMS scoring system are commonly collected in these studies. In two of four studies, AMS severity was found to increase in HH compared with NH [14,16]. Furthermore, in the one study that found no differences between environments [6], participants only had a 5-min exposure time, as opposed to 9- and 10-h exposures in other studies. Additionally, postural stability was significantly worse in HH [23]. The authors suggest that visual contrast sensitivity is lower in HH than in NH and that this may contribute to the postural stability [23].

#### Other homeostatic variables

Plasma pH appears to be higher in HH than NH [17,18,22]. The study [20] that did not find significant differences in pH between the two conditions was conducted at 1,829 and 2,438 m whereas the elevated pH values were from 4,500 m, suggesting that the differences between the two conditions may be more pronounced at higher altitudes. Elevated pH in HH is surprising in the context of the finding that ventilation is reduced under these conditions.

Exhaled and systemic NO levels were lower in HH [12,19]. Faiss et al. [12] found increased oxidative stress in HH and attributed the systemic differences in NO bioavailability to this.

Many other physiological variables were measured; however, most of these were reported in only a single study. Thus, it is difficult to make conclusions without verification from other studies, and we have not considered these further.

### Study quality

The search results reveal several issues relating to study design. Very few studies state the reliability of their measurements or performed a sample size calculation. It is therefore difficult to evaluate if they are adequately powered to identify a real difference between conditions, should such a difference exist. Given that differences in physiological responses between NH and HH conditions are likely quite small, large sample sizes would likely be required to avoid type II (false negative) errors. Additionally, by performing statistical analysis on a large number of variables over many time points, the risk of type I (false positive) errors increases.

Failure to account for the P_H2O_ leads to an overestimate of the hypoxic dose in NH, such that incorrect partial pressure of inspired oxygen (PiO_2_) may be attained [11]. In one study [6], a NH exposure equivalent to 7,620 m was described; however, the conditions were in fact closer to 7,010 m once pH_2_O was accounted for [11]. We have emphasised these differences by calculating, where possible, the PiO_2_ in the different hypoxic conditions (Table 2: Study design). We found the differences in PiO_2_ to be as much as 4 mmHg. The level of CO_2_ in the test environment was a potential source of error. Basualto-Alarcon et al. [21] highlight this issue in stating that different gas inflow rates into each hypoxic system allow different levels of CO_2_ accumulation. Additionally, they state that their NH environment may have been more hypercapnic because it had half the total volume of the hypobaric chamber. These control group contrast issues will either enhance or diminish the effect size and therefore the difference between HH and NH. This may be of particular relevance to ventilator variables.

### Mechanisms for results

Many hypothetical mechanisms have been proposed for the effect of low barometric pressure on physiology. These include intravascular bubble formation, increased alveolar deadspace, altered fluid permeability, changes in chemosensitivity, and a mismatch in ventilation and perfusion [13,16,17]. Although pressure may be the principle confounder between the two scenarios, we must also reflect that other factors may differ between HH and NH, thus impacting participant's physiology. For example, the laboratory-based components of the studies reviewed were performed between 22°C and 25°C, a temperature likely to be far warmer that experiences at 4,000 m in a field laboratory. Such differences in ambient temperature may alter physiological mechanisms such as the degree of peripheral vasoconstriction, NO metabolism or the production of reactive oxidative species [24].

As highlighted, the duration of the hypoxic exposure impacts on the results obtained. Different physiological systems will have different response rates for adaptation to hypoxia [25]. For some physiological parameters, the short study durations may not be long enough for differences between NH and HH to be elicited. Studies reporting repeated measures over time provide a window on this phenomenon. For example, in the 1997 study by Loeppky et al. [13], where no differences in minute ventilation were reported after 30 or 60 min of hypoxic exposure, significant differences were evident after 3 h of exposure. Additionally, Savourey et al. [17,18] initially found lower P_ET_O_2_ and P_ET_CO_2_ in HH than NH but then no difference in prolonged exposure. This may be because during HH exposure, the ambient partial pressure of nitrogen (P_N2_) is initially lower than the body's and therefore nitrogen (N_2_) initially diffuses from the tissues to the alveoli [5]. Until this equilibrium is achieved, the arterial oxygen content, P_A_O_2_, and the arterial carbon dioxide content, P_A_CO_2_, are lowered as a result of the relatively higher P_A_N_2_ in HH than NH.

Loeppky et al. [13] also suggests that an initial increase in CO_2_ produced in HH compared to NH might be due to microbubble formation similar to the nitrogen bends in divers. This emphasises the importance of study duration on physiological response and the problems inherent in comparing studies of different hypoxia exposure times.

If there are indeed differences between HH and NH, at what equivalent altitude do they become apparent? Most of the studies have been carried out at 4,500 m (or equivalent), but Naughton et al.'s study [20] performed at 1,829 and 2,438 m was unable to find any significant differences between HH and NH. These altitudes correspond to PO_2_ values of 118 mmHg (15.7 kPa) and 108 mmHg (14.4 kPa) [25] respectively, and it is possible that these altitudes were not high enough to elicit differences in the measured variables. Significant differences between the effects of NH and HH may impact the interpretation and application of results from studies at high altitude where the change in pressure may be a confounding influence in the evaluation of physiological responses to high altitude.

### Strengths and limitations of this study

Although this is the first systematic review to summarise crossover studies comparing physiological responses to hypobaric and normobaric hypoxia, other publications have come to similar conclusions on the topic. Millet et al. [10] stress the importance of disentangling hypoxia and hypobaria and Fulco et al. [26] highlight the need for further investigations into NH versus HH, for particular application to pre-acclimatisation strategies.

The strengths of this systematic review include the clear research question, comprehensive search strategy and consistent methods used for identifying eligible manuscripts and extracting data. Limitations of this review include the focus on crossover studies but are predominantly related to the quantity and quality of the underlying literature. There are few studies that compare HH and NH and the number of participants in each study is small. Whilst several of these studies report interesting differences between HH and NH, there is marked inconsistency in the reported results. This may be due to a number of other factors including heterogeneity of study design, duration and magnitude of hypoxic dose and outcome reporting. Furthermore, the reporting of multiple phenotypes in each study without correction for repeat testing may be associated with an increased likelihood of type 1 errors. Conversely, the small sample sizes may be associated with an increased likelihood of type 2 errors.

As mentioned, the studies were heterogeneous by design. For example, they differed in regard to the subjects' prior exposure to altitude. In two of the studies reviewed [16,22], the subjects lived between 1,500 and 1,600 m and so may have been partially acclimatised to high altitude. It is not clear whether the same effects would be seen in partially and not acclimatised subjects.

Finally, the self-reported nature of AMS scores could be associated with inconsistent responses from participants. In the study by Self et al. [6], there was a disparity between post-hypoxia interview responses and the responses during hypoxic exposure. There is no gold standard method for these types of studies and so there is much variability due to the methodology employed.

## Conclusions

We present an overview of the current available literature regarding crossover studies relating to the different effects of HH and NH on human physiology. This systematic review is the first to compare the effects of a NH and HH environment on human physiology. Several studies reported a number of variables (e.g. minute ventilation and NO levels) that were different between the two conditions, lending support to the notion that true physiological difference are indeed present. However, the presence of confounding factors such as time spent in hypoxia, temperature, and humidity, and the limited statistical power due to small sample sizes, limit the conclusions that can be drawn from these findings.

Standardisation of study methods and reporting may aid interpretation of future studies and thereby improve the quality of data in this area. This is important to improve the quality of data that is used both for improving understanding of hypoxia tolerance, both at altitude and in the clinical setting.

## Abbreviations

ACTH: Adrenocorticotropic hormone
ADH: Anti-diuretic hormone
AMS: Acute mountain sickness
ANP: Anti-naturetic protein
Bf: Respiratory rate
BP: Blood pressure
CO_2_: Carbon dioxide
CO: Cardiac output
ECG: Electrocardiogram
FetCO_2_: End tidal fraction of carbon dioxide
FetO_2_: End tidal fraction of oxygen
exNO: Exhaled nitric oxide levels
GFR: Glomerular filtration rate
GPX: Glutathione peroxidase
Hb conc: Haemoglobin concentration
Hct: Haematocrit
HR: Heart rate
LF/HF: Heart rate variability
pH_2_O: Humidity
HH: Hypobaric hypoxia
HCR: Hypoxic cardiac response
HVR: Hypoxic ventilatory response
NO: Nitric oxide
N_2_: Nitrogen
NH: Normobaric hypoxic
SpO_2_: Pulse oximetry
P_N2_: Partial pressure of nitrogen
PO_2_: Partial pressure of oxygen
PaCO_2_: Arterial partial pressures of carbon dioxide
PaO_2_: Arterial partial pressures of oxygen
PetO_2_: End tidal partial pressure of oxygen
P_ET_CO_2_: End tidal partial pressure of CO_2_
Plasma K^+^: Plasma potassium concentration
plasma Na^+^: Plasma sodium concentration
RQ: Respiratory quotient
SaO_2_: Arterial blood saturations
SV: Stroke volume
urine Na^+^/K^+^: Urine sodium-potassium ratio
VA: Alveolar ventilation
VCO_2_: Volume of CO_2_ produced
VE: Ventilation
VO_2_: Volume of oxygen consumed
VT: Tidal volume
